# Hydatid, or Encysted Disease of the Testicle

**Published:** 1831-01-01

**Authors:** 


### XXXII.

### HYDATID OR ENCYSTED DISEASE OF THE TESTICLE.

IN our review of Sir Astley Cooper's book, we have necessarily been compelled to omit many of the cases, which he relates in illustration of his doctrines. We shall take the opportunity of noticing them in detached articles in this department of the Journal. We commence with the hydatid or encysted disease, premising, however, that the term hydatid is not meant to imply the existence of true hydatids in this affection. As these parasitic animals are sometimes met with in the testis, we think it would be better to limit the appellation of hydatid disease to that in which they really exist. The other might simply be named the encysted.

*Case* 1. A young man, 20 years of age, found a tumour, without obvious cause, at one extremity of the testis, but at which he could not say. It was at first unattended with pain—was usually soft—but, as it grew more and more, it became, though generally soft, at times extremely hard. It was now extremely painful on some occasions, but commonly there was little uneasiness. After a twelvemonth it was extracted, and the spermatic cord being tied too loosely, exuberant granulations sprouted from its extremity and required a second and tighter ligature. On cutting into the testicle it was found to be composed of numerous cysts, of different sizes and forms, containing a fluid like serum in some parts, like white of egg in others. In one part the testis was very compact, and at that part there was a tendency to suppuration.

*Case* 2. A young medical man consulted Sir Astley with an enlargement of the testis, which was about seven times its natural size. It was quite unattended with pain—its increase had been very gradual—its weight was considerable—its fluctuation was very obscure—and it was not transparent. His general health was good and Sir Astley recommended the operation, which was done by Mr. Guthrie. The disease on examination proved to be of the encysted kind. The patient gradually recovered and did very well.

*Case* 3. Bartholomew Lupre, ætatis 30, an Italian sailor, was admitted into Guy's Hospital in April, 1809, with an enlarged testis, which he reported had begun four or five months before his admission. Its cause was unknown, but he attributed it to wearing wet clothes. The veins of the scrotum were very large, and those of the cord were varicose. There was also much pain in the loins from the weight of the swelling. Sir Astley performed the operation, and found the disease to be of the encysted kind. The result is not mentioned, but we suppose it was favourable.

---

